# Effects of Epinephrine on Inflammation-Related Gene Expressions in Cultured Rat Cardiomyocytes

**Published:** 2017

**Authors:** Stacey Chen, Geoffrey L. Liu, Marilyn M. Li, Renyu Liu, Henry Liu

**Affiliations:** 1 Department of Surgery, New York University Langone medical Center; 2 Division of Genomic Diagnostics, Department of Pathology & Laboratory Medicine, Children’s Hospital of Philadelphia, Perelman School of Medicine at the University of Pennsylvania; 3 Department of Anesthesiology and Critical Care, Perelman School of Medicine at the University of Pennsylvania; 4 Department of Anesthesiology & Perioperative Medicine, Drexel University College of Medicine, Hahnemann University Hospital

**Keywords:** Epinephrine, Gene expression, Cardiomyocyte, Inflammation

## Abstract

Epinephrine, a non-specific adrenergic agonist, is one of the most commonly used inotropes perioperatively. Recent studies have shown that inflammatory response in cardiac surgery could result in hypoperfusion, dysrhythmias, myocardial ischemia, and other pathophysiological alterations in the postoperative period. These alterations might be contributing to the adverse clinical outcome. Although epinephrine has been shown to have effects on the immune system, how epinephrine affects inflammatory response is unclear. We hypothesized that epinephrine exposure may alter the inflammatory response which may potentially contribute to the adverse clinical outcomes. We used cultured rat cardiomyocytes (H9C2) with epinephrine exposure in this study. The expression of mRNA for inflammation-related genes was quantitated for the comparison of experimental group (with epinephrine) and control group (without epinephrine). The results demonstrated significant changes of inflammation-related gene expressions in cardiomyocytes after epinephrine administration. The clinical implications of the gene expression changes in cardiomyocytes are unclear.

## Introduction

Heart failure is a clinical syndrome characterized by either a structural or functional cardiac abnormality that results in the inability of the heart to adequately maintain cardiac output (CO) and subsequent blood circulation to meet the metabolic demands of the human body [1, 2]. Neurohumoral adaptations, such as activation of the sympathetic nervous system, renin-angiotensin-aldosterone system, and increased secretion of antidiuretic hormone and natriuretic peptides serve as compensatory mechanisms to maintain CO and perfusion to vital organs [1, 2]. However, while these mechanisms are essential to preserving the physiologic function of the heart and satisfy the metabolic needs in the early stages of pathophysiologic change, as the disease progresses, these adaptations can become maladaptive and contribute to a vicious cycle of worsening symptoms of heart failure [2]. Sympathetic activation (epinephrine release) in heart failure patients results in enhanced ventricular contractility and higher heart rate which function to maintain patient’s CO. Epinephrine at lower doses predominately acts on β1-adrenergic receptors distributed mainly in cardiomyocytes, and results in an increase in CO with a decrease in SVR [3, 4]. Epinephrine at higher doses will have potent effects on α-adrenergic receptors, producing not only an increase in CO, but also an increase in SVR, which can potentially worsen pre-existing myocardial workload and coronary ischemia, counteracting its initial hemodynamic beneficial effects [5]. Furthermore, in the setting of cardiac stress status (i.e. hypoxia, ischemia, hypertrophy), there is certain degree of cardiac remodeling resulting in the induction of apoptosis.

Patients with heart failure often have a low CO status. They are at increased risk for cardiac surgery to develop major complications perioperatively. Their low CO status often necessitates inotropic support to maintain hemodynamic stability [6]. Among positive inotropic agents available, epinephrine is one of the most commonly used perioperatively. Epinephrine stimulates production of the second messenger 3’–5’-cyclic adenosine monophosphate (cAMP) [7]. However, studies have indicated that the use of epinephrine does not seem to improve longer term clinical outcomes, as its mechanism of action would theoretically suggest, and may even increase postoperative mortality and morbidity [8, 9, 10]. Current studies have shown that one explanation for this is that while epinephrine maintains cardiac output, it does so at the expense of producing an increased risk of tachycardia and other arrhythmias, ischemia, and lactic acidosis in the postoperative state. Cardiac surgery induces a vigorous stress and inflammatory response in the body, which results in perioperative metabolic changes such as increased oxygen consumption and energy expenditure, increased secretion of epinephrine, norepinephrine, cortisol, adrenocorticotropic hormone (ACTH), insulin and growth hormone. Perioperative inflammatory responses and/or systemic inflammatory response syndrome (SIRS) with activation of the coagulation, complement, kallikrein, and fibrinolytic cascades may contribute significantly to perioperative morbidities and mortalities [11, 12]. We hypothesize that epinephrine exposure in cardiomyocytes may alter gene expressions related to inflammation, which subsequently leads to potential adverse clinical outcomes.

## Materials and Methods

The details of our method were described in our previous publication [13]. Briefly, we used H9C2 rat cardiomyocyte cell-line (ATCC, Rockville, Maryland) in our study. The H9C2 cardiomyocytes were inoculated in 25ml flask (Therma Fisher Scientific, Waltham, MA USA) to the final concentration of 0.5M/mL. The cells were cultured at 37°C in DMEM with 10% fetal calf serum. These cardiomyocytes then were given time to settle down overnight. Epinephrine was added to reach the final concentration at 1 µM in the culture media. The H9C2 cells without exposing to epinephrine served as controls. After 24 hours, RNA was extracted from the cultured cardiomyocytes and prepared for whole genome gene expression study with microarray. This microarray contains 41,000+ rate genes, and cDNA was synthesized from RNA samples and then used to synthesize fluorescent cRNA. Labeled cRNA samples were hybridized to the Whole Rat Genome Oligo Microarray slides. Microarrays were washed and scanned after hybridization. All arrays were run in triplicate. These experimental data were then imported into the GeneSpring software as 20 one-color arrays and normalized to the median per chip and to the median value per gene across all microarrays. Parameter data were also added so that microarrays could be grouped by the time and treatment. Guided workflow returned reasonable amount of gene lists. These were analyzed for significant Gene Ontology and pathway hits based on passed P value (< 0.05 is used as the cut off).

## Results

**Figure 1:**
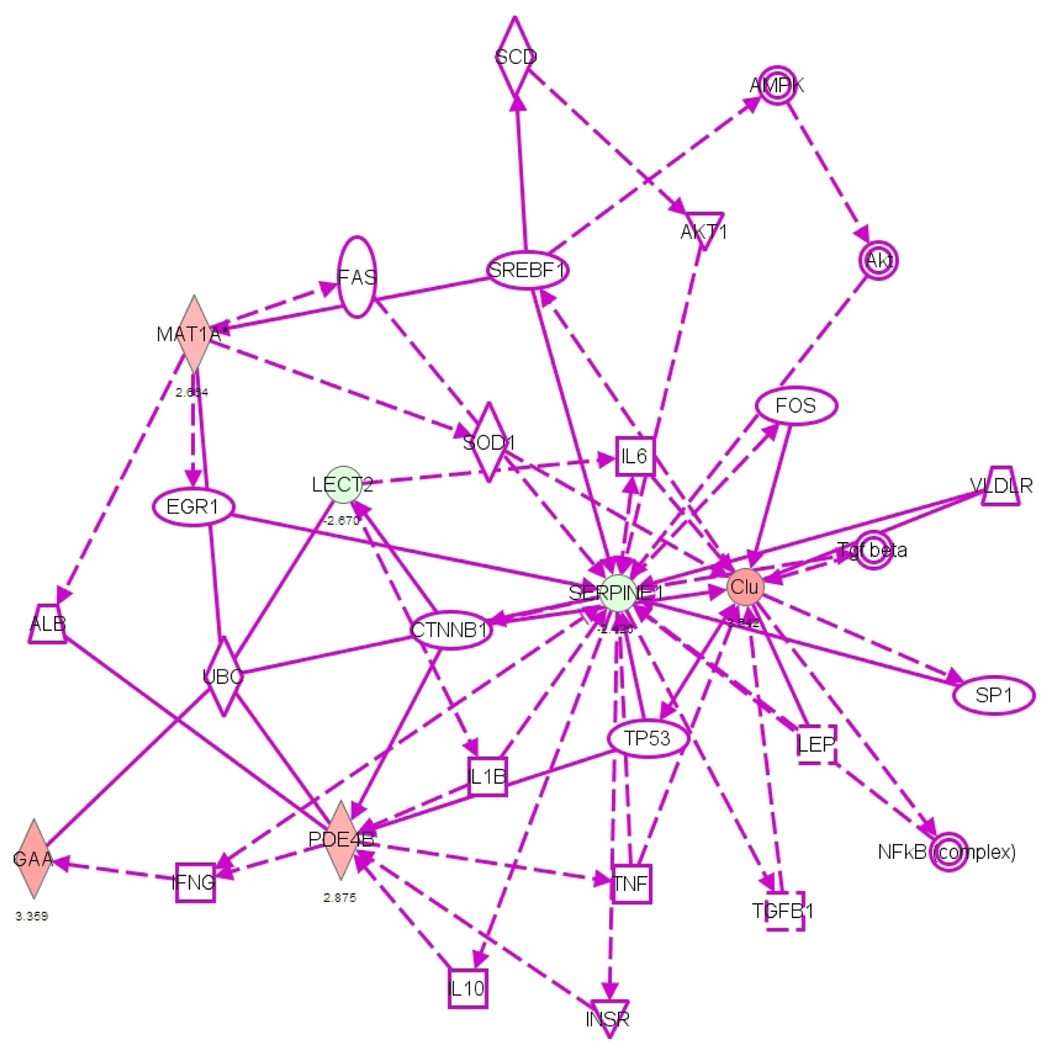
Scheme indicating the gene expression changes related to inflammation after exposure of cardiomyocytes to epinephrine (Green indicates decreased gene expression, red indicates increased expression). CLU= Clusterin, MAT1A= methionine adenosyltransferase 1α, GAA= lysosomal α-glucosidase, LECT2= chemotaxin 2, SERPINE1/PAI-1= plasminogen activator inhibitor-1, PDE4= phosphodiesterase isoenzymes-4.

Exposure of cultured cardiomyocytes to epinephrine induced significant gene expression changes. The following gene expressions were upregulated: Clusterin’s gene expression (CLU) increased by 3.84 times, methionine adenosyltransferase 1α (MAT1A) 2.66 times, lysosomal α-glucosidase (GAA) 3.36 times and phosphodiesterase isoenzymes PDE4 2.88 times. The downregulated gene expressions included chemotaxin 2 (LECT2) decreased by −2.67 times and plasminogen activator inhibitor-1 (SERPINE1/PAI-1) decreased by −2.42 times. The inflammation-related gene expression changes induced by epinephrine exposure were shown in Table 1 and Figure 1.

## Discussion

Epinephrine is a non-specific adrenergic agonist and one of the most commonly used positive inotropic agents in the perioperative management of patients with heart failure and/or low cardiac output syndrome undergoing cardiac or non-cardiac surgery. In this study, we investigated the gene expression changes related to inflammation in cultured cardiomyocytes exposed to epinephrine. Our study identified four epinephrine-induced up-regulated gene expressions and two down-regulated gene expression changes related to inflammation.

The effects of epinephrine on cells are mediated by its interactions with adrenergic receptors to increase intracellular levels of cAMP, which will in turn produce a wide variety of biochemical responses depending upon which tissues are affected [7]. In cardiomyocytes, increased cAMP activates a signaling cascade that will ultimately result in increased calcium entry into cardiomyocytes and subsequently increased contractility. Intracellular cAMP levels are regulated by phosphodiesterase isoenzymes PDE3 and PDE4 [14]. Our study showed increased PDE4B gene expression in rat cardiomyocytes after exposure to epinephrine. Galindo-Tovar *et al* used a porcine heart model to study the interaction of PDE3 and PDE4 with cardiac β1- and β2-adrenergic receptors in different regions of the heart and concluded that while both PDE3 and PDE4 reduce the inotropic effects produced by epinephrine via its interaction with β2 receptors in the atria [15]. The up-regulated PDE4B gene expression could theoretically lead to further reduction of the inotropic effect of epinephrine, which may imply that upregulation of PDE4 gene expression may be contributing to epinephrine’s gradual decline of inotropic effect.

Interestingly, recent studies have demonstrated that lymphocytes and phagocytes not only express adrenergic receptors on their cell surfaces, but are also capable of synthesizing and secreting the catecholamines (epinephrine and norepinephrine) [16, 17]. PDE4 is one of the most abundant isoenzymes in inflammatory cells. Delgado *et al* showed a time- and concentration-dependent relationship between epinephrine and PDE4 in a monocytic cell line exposed to epinephrine [18]. This correlation found in both cardiomyocytes and monocytes supports the concept that catecholamines play an important role in the communication between the endocrine and immune systems.

In patients with heart failure, there is a selective reduction in β1-receptors or/and receptor reactivities while the expression of β2 receptors is relatively unchanged [19]. The β1-receptors promote apoptosis via cAMP-dependent mechanism [20]. Epinephrine works on β1-receptors as agonist to possibly induce more apoptosis theoretically. Our study showed rat cardiomyocytes treated with epinephrine had increased expression of the CLU gene, which encodes Clusterin. Clusterin exists as three isoforms: secretory (extracellular form, sCLU), cytoplasmal (cCLU), and a nuclear form (nCLU). Functioning as a chaperone, sCLU prevents protein aggregation and promotes overall cell survival [21]. Kim *et al* reported that overexpression of sCLU resulted in a down-regulation of inflammatory chemokines and cell adhesion molecules, which indicated possible negative impact on inflammatory responses [22]. And nCLU has been reported promotion of cell death via apoptosis; upregulation of nCLU gene expression will likely induce more apoptosis and cell death. The gene expressions for methionine adenosyltransferase 1α (MAT1A) and lysosomal α-glucosidase (GAA) were also upregulated in our study. MAT1A catalyzes a two-step reaction that produces S-adenosylmethionine and tripolyphosphate. S-adenosylmethionine serves as a principal methyl donor [23]. The MAT1A isoform is specific to the liver and it is unknown what is the significance of its upregulation in the presence of epinephrine and how it mediates its effects in inflammation. The GAA gene encodes lysosomal α-glucosidase, an enzyme essential for the metabolism of glycogen to glucose. In addition to its myocytic and vascular effects, epinephrine induces metabolic pathways such as increasing glucose, glycerol, and fatty acid production [11]. Liao *et al* also demonstrated that hyperglycemia contributes to cardiac hypertrophy and heart failure. They were also able to show that glycemic control with the α-glucosidase inhibitor voglibose reduces the severity of heart failure via inhibition of NADPH oxidase in murine animal models [24]. The upregulation of GAA stimulated by epinephrine potentially results in an increase in hyperglycemia-induced inflammation.

One of the effects of cardiopulmonary bypass is SIRS, which is characterized by tachycardia, tachypnea, fever or hypothermia, leukocytosis or leukopenia [25, 26]. The extracorporeal circulation induces laminar blood flow, endothelial activation with subsequent upregulation of chemokines, leukocyte adhesion proteins, and pro-coagulation proteins, and oxidative stress, which results in reperfusion injury, release of pro-inflammatory cytokines, endotoxemia, and activation of the complement system, coagulation cascade, leukocytes and platelets that all serve as contributing factors to the development of SIRS. One of the relatively newly identified chemotactic factors, leukocyte cell-derived chemotaxin 2 (LECT2), has been implicated as a possible marker for trending the inflammatory process. Ando *et al* showed that plasma levels of LECT2 in septic patients who had been admitted to ICU were significantly lower compared to healthy volunteers who served as controls and the LECT2 levels had significantly increased in these septic patients at the time of ICU discharge, suggesting that LECT2 is involved in the suppression of excessive inflammation [27]. This is further supported by the fact that LECT2 has also been shown to inhibit the production of pro-inflammatory cytokines such as TNF-α, IL-1β, and IL-6 in murine models [28]. In our study, gene expression of LECT2 was downregulated in epinephrine treated cardiomyocytes. The downregulation of LECT2 in the presence of epinephrine suggests that epinephrine may have a proinflammatory role and contributes to tipping the balance towards SIRS in patients undergoing cardiac surgery with cardiopulmonary bypass.

Our study also demonstrated decreased gene expression of plasminogen activator inhibitor-1 (SERPINE1/PAI-1). PAI-1 encodes a serine protease inhibitor of tissue plasminogen (tPA) and urokinase (uPA), which are the activators of fibrinolysis. Tissue plasminogen and urokinase cleave inactive plasminogen to plasmin and the plasmin will in turn degrade fibrin or fibrinogen for degradation [29]. Thus, PAI-1 mediates hypercoagulable states and thrombosis and studies have suggested that the release of PAI-1 in response to stress and elevated levels of PAI-1 are associated with the development of coronary artery disease [30]. In inflammatory conditions where fibrin is deposited in tissues, PAI-1 appears to play a significant role in the progression to fibrosis during inflammatory event. Downregulation of SERPINE1/PAI-1may suggest the inhibition of inflammatory condition-associated fibrosis.

The clinical utilization of epinephrine offers significant short term hemodynamic benefits, including improvement of myocardial contractility leading to better cardiac output, mean arterial blood pressure. But these benefits may come at the expense of potential longer term adverse clinical outcome. In this study, we demonstrated that epinephrine exposure could induce inflammation- related gene expression changes in cultured rat cardiomyocytes. These gene expression alterations may have direct effect on inflammatory responses or play roles in the regulation of inflammatory processes. However the exact clinical implications of these gene level changes are unclear, further investigations will be needed. In vivo animal experiments and human studies will help validate the findings and illustrate their clinical significance.

In conclusion, the results from our study showed significant changes of inflammation-related gene expressions in cultured rat cardiomyocytes treated with epinephrine. The clinical implications of these gene expression changes are unclear, further investigations will be needed.

## Figures and Tables

**Table 1:** Epinephrine-induced gene expression changes

Up-regulated genes	CLU	+3.84
	GAA	+3.36
	PDE4B	+2.88
	MAT1A	+2.66

Down-regulated genes	LECT2	−2.67
	SERPINE1	−2.42

CLU: Clusterin; MAT1A: methionine adenosyltransferase 1A; GAA: lysosomal α-glucosidase; PDR4: phosphodiesterase isoenzymes; LECT2: chemotaxin 2; SERPINE1/PAI-1: plasminogen activator inhibitor-1.
